# Immuno-informatics design of a multimeric epitope peptide based vaccine targeting SARS-CoV-2 spike glycoprotein

**DOI:** 10.1371/journal.pone.0248061

**Published:** 2021-03-17

**Authors:** Onyeka S. Chukwudozie, Clive M. Gray, Tawakalt A. Fagbayi, Rebecca C. Chukwuanukwu, Victor O. Oyebanji, Taiwo T. Bankole, Richard A. Adewole, Eze M. Daniel

**Affiliations:** 1 Department of Cell Biology and Genetics, University of Lagos, Lagos, Nigeria; 2 Division of Immunology, Institute of Infectious Disease and Molecular Medicine and Department of Pathology, University of Cape Town, Cape Town, South Africa; 3 Immunology Unit, Medical Laboratory Science Department, Nnamdi Azikiwe University, Nnewi, Nigeria; 4 Department of Veterinary Pathology, University of Ibadan, Ibadan, Nigeria; 5 Public Health Biotechnology Unit, Institute of Child Health, University College Hospital, University of Ibadan, Ibadan, Nigeria; Frederick National Laboratory for Cancer Research, UNITED STATES

## Abstract

Developing an efficacious vaccine for SARS-CoV-2 infection is critical to stemming COVID-19 fatalities and providing the global community with immune protection. We have used a bioinformatic approach to aid in designing an epitope peptide-based vaccine against the spike protein of the virus. Five antigenic B cell epitopes with viable antigenicity and a total of 27 discontinuous B cell epitopes were mapped out structurally in the spike protein for antibody recognition. We identified eight CD8+ T cell 9-mers and 12 CD4+ T cell 14-15-mer as promising candidate epitopes putatively restricted by a large number of MHC I and II alleles, respectively. We used this information to construct an *in silico* chimeric peptide vaccine whose translational rate was highly expressed when cloned in pET28a (+) vector. With our *In silico* test, the vaccine construct was predicted to elicit high antigenicity and cell-mediated immunity when given as a homologous prime-boost, triggering of toll-like receptor 5 by the adjuvant linker. The vaccine was also characterized by an increase in IgM and IgG and an array of Th1 and Th2 cytokines. Upon *in silico* challenge with SARS-CoV-2, there was a decrease in antigen levels using our immune simulations. We, therefore, propose that potential vaccine designs consider this approach.

## Introduction

An unprecedented pneumonia disease outbreak was reported in late December 2019, after several deaths were recorded in Wuhan, China **[1]**. There was a rapid spread of the disease from the city of Wuhan to many countries, including the United States, with thousands infected and many dying within months of initial spread **[1, 2]**. On December 31, 2019, the disease outbreak was traced to Coronavirus’s novel strain **[1]**. It was later termed SARS-CoV-2 and the disease COVID-19 by the WHO **[3, 4]**. Reports showed that as of June 16, 2020, at least 440,421 confirmed deaths and more than 8,144,359 confirmed cases, and the current statistics as of November 27 are currently at 1.4 million deaths with 61.2 million cases of infection **[1, 5]**. The SARS-CoV-2 has been identified as a new strain from group 2B Coronaviruses, with approximately 70% genetic similarity to SARS-CoV, from the 2003 outbreak **[4]**. The virus has a 96% similarity to a bat coronavirus, so it is widely suspected to originate from bats **[6, 7]**. The pandemic has resulted in travel restrictions and nationwide lockdowns in several countries and resulted in economic mayhem **[1]**. Genome sequences have been deposited in several curated online biorepositories in the search for solutions and developing a universally available vaccine is critical.

Coronaviruses are a group of viruses that generally causes disease in mammals and Aves. In humans’ case, coronaviruses are responsible for respiratory tract infections that can range from mild, such as cases of the common cold and low fever, and others that can be lethal, such as SARS, MERS, and COVID-19 **[4]**. Cases have shown that symptoms can vary depending on the host; for example, upper respiratory tract disease has been found in chickens and diarrhea in cows and pigs **[6]**. The Coronavirus is grouped under the subfamily *Orthocoronavirinae*, in the family Coronaviridae, order Nidovirales **[5, 6]**. They are positive-sense single-stranded RNA viruses with a nucleocapsid of helical symmetry. They are also enveloped with spike proteins and the genome size of many of the coronaviruses range from 27–34 kilobases, the largest among known retroviruses **[8]**. The current zoonotic jump to humans is of concern as the upper respiratory tract infection can lead to fatal disease in some individuals. There is yet to be an effective vaccine to prevent or treat any human coronavirus infections **[4]**.

Structural analysis has revealed that the Spike protein S1 facilitate the attachment of the virion to the cell membrane by interacting with both the ACE2 and CLEC4M/DC-SIGNR receptors **[9]**. A conformational change of the S glycoprotein is induced at the viral pathogen’s internalization into the endosomal compartment of the host **[9]**. Proteolysis by group of lysosomal cathepsin L families may unmask the fusion peptide of S2 and activate membranes fusion within host endosomes. The spike protein S2, which acts as a class I viral fusion protein, mediates the fusion of the virion and cellular membrane. There are three conformational attributes of the protein: pre-fusion native state, pre-hairpin intermediate, and post-fusion hairpin state **[9]**. During viral and target cell membrane fusion, the coiled regions (heptad repeats) assume a trimer-of-hairpin structure, positioning the fusion peptide close to the C-terminal region of the ectodomain **[10]**. This structural formation facilitates the subsequent fusion of the viral protein and its target cell membrane **[10]**. Upon endocytosis, the spike protein S2 acts as viral fusion peptide and it is unmasked following S2 cleavage occurring upon virus endocytosis **[10]**. The receptor-binding domain (RBD) on the spike protein coupled with the cellular ACE2 receptor which characterizes part of the molecular mechanism surrounding the viral entry and infection has been identified **[11, 12]**. Studies have shown that the induction of neutralizing antibodies and angiotensin-converting enzyme 2 (ACE2) binding process that aids in the viral entry are mediated by the RBD of the S1 region of the S protein **[13]**. Experimental studies have also shown that the continuous depletion of the RBD-antibodies from sera components reduced serum-neutralizing capability, indicating that this domain is dominant in neutralizing antibody induction **[14]**. The S protein’s pivotal role in viral infection has made it a top candidate for vaccine production. There are currently over 90 SARS-CoV-2 vaccine candidates **[1]**, and an epitope-based vaccine may provide a useful complimentary approach that would steer immunity to immunogenic epitopes on the S protein. With the immuno-informatics, it is now possible to evaluate proteins’ immunogenic properties via computational methods (*in silico*) with high efficiency **[15–17]**. We have therefore used the *In silico* checkpoint to design an epitope peptide-based vaccine against the SARS-CoV-2 spike glycoprotein and also mimic the range of responses in a prime-boost scenario.

## Materials and methods

### Data retrieval, structural and physiochemical analysis of SARS-CoV-2 spike protein

The protein sequence from different geographical regions was retrieved from the NCBI repository with their corresponding accession numbers: Wuhan, China (Genbank ID: QHD43416.1), Japan (Genbank ID: BCA87361.1), California, USA (Genbank ID: QHQ71963.1), Washington, USA (Genbank ID: QHO60594.1), and Valencia, Spain (Genbank ID: QIQ08790.1). The protein structure of the SARS-CoV-2 spike (PDB: 6VSB) was downloaded from the protein data bank. The physiochemical properties of the protein sequence such as the GRAVY (Grand average of hydropathicity), half-life, molecular weight, instability index, aliphatic index, and amino acid atomic composition was bio-computed via an online tool Protparam (http://web.expasy.org/protparam/) **[18]**.

### Prediction of B cell linear and discontinuous epitopes

The Bepipred server from the Immune-Epitope-Database and Analysis-Resource (IEDB) database was used for this prediction and to identify putative B cell linear epitopes **[19]**. Bepipred-2.0 executes its operation based on a random forest algorithm trained on epitopes annotated from antibody-antigen protein structures **[19]**. This method is superior to other available tools for sequence-based epitope prediction with regard to both epitope data derived from solved 3D structures and an extensive collection of linear epitopes downloaded from the IEDB database **[19]**. The following criteria, such as the specificity at 75% and 14–15 mers (residues), binds to various MHC alleles. Several conditions such as antigenicity, accessibility of surface, flexibility, hydrophilicity is imperative for predicting the B cell linear epitopes. These conditions are considered when making predictions with the Bepipred linear epitope prediction and Parker hydrophilicity prediction algorithms.

We used the SVMTriP (http://sysbio.unl.edu/SVMTriP/) in the prediction of the B cell linear epitopes. The SVMTriP is a Support Vector Machine method used to predict linear antigenic epitopes, which combine the Tri-peptide similarity and Propensity scores (SVMTriP). Application of SVMTriP to non-redundant linear B-cell epitopes extracted from IEDB achieved a sensitivity of 80.1% and a precision of 55.2% with five-fold cross-validation. Predicted epitopes were subjected to Vaxijen 2.0 for antigenicity test **[20]**. We further predicted the discontinuous epitopes that had a more significant attributes than the linear epitopes. The discovery of discontinuous B-cell epitopes is a considerable challenge in vaccine design. Previous epitope prediction methods have mostly been based on protein sequences and are not particularly useful. Therefore, the DiscoTope server was used to predict the surface accessibility and amino acids that form discontinuous B cell epitopes found from X-ray crystallography of antigen/antibody protein buildings. We utilize the Pymol visualization software to examine forecast epitopes’ positions on the 3D structure of SARS-CoV-2 protein **[21]**.

### Prediction of epitopes restricted by class I Human Leukocyte Antigen (HLA) CD8+ (CTL) and class II HLA CD4+ T cells (HTL)

For *de novo* prediction of Covid-19 spike glycoprotein CD8+ T cell epitopes (peptides), we used IEDB MHC I binding prediction algorithms (http://tools.iedb.org/mhci). This method integrates the epitopes’ prediction restricted to a large number of MHC class I alleles and proteasomal C- terminal cleavage, adopting complex artificial neural network applications. For accuracy, other software such as an artificial neural network (ANN), stabilized matrix method (SMM), MHC binding energy covariance matrix (SMMPMBEC), NetMHCpan, pickpocket, and NetMHCstapan, were adopted for this purpose. All of these predictive tools are archived on the IEDB (Immune-Epitope-Database and Analysis-Resource) database with a mathematical threshold before best-fit epitopes are selected from each online server. To predict the CD4+ T cell epitopes (peptides), we used the MHC II binding predictions tool (http://tools.iedb.org/mhcii/) found in the IEDB database. First, we selected the epitopes whose binding diversities with the different HLA serotypes were higher. We further subjected these epitopes to the Vaxijen 2.0 server to test their antigenicity at a recommended threshold of 0.7. We also considered for further analysis by subjecting the top-scoring predicted epitopes from each tool that have been predicted by five or more different methods and submitted them to IEDB T cell Class I Immunogenicity predictor (http://tools.iedb.org/immunogenicity/). Results were obtained in descending score values. However, the table can also be sorted by clicking on individual column headers. The higher score indicates a greater probability of eliciting an immune response.

### Profiling of the selected T cells epitopes

Following the selection of HLA-restricted CD8+ and CD4+ T cell epitopes, critical features such as peptide toxicity predicted from the ToxinPred server (http://crdd.osdd.net/raghava/toxinpred/), allergenicity, indicated from AllergenFP 1.0, and digestion predicted from protein digest server were made. All of these criteria were considered before the final selection of the T cell epitopes. Epitopes with no peptide toxicity attributes were selected. Antigenicity testing was conducted through the Vaxijen v2.0 server (http://www.ddgpharmfac.net/vaxijen/VaxiJen/VaxiJen.html) **[20]**, which operate based on auto- and cross-covariance transformation of the input protein sequence into uniform vectors of principal amino acid properties. The antigenicity index was generated at a threshold of 0.7.

### Epitope conservancy in related SARS-CoV-2 spike protein from different geographical locations

Conservation analysis of selected epitopes is the fraction of a protein sequence that contains the epitope, while the identity is the degree of correspondence (similarity) between the sequences. We computed the degree of epitope conservancy within the SARS-CoV-2 spike glycoprotein sequence and set at a given identity level of 100 using the IEDB conservation-analysis-tool.

### The HLA-A 02*01 allelic affinity of the CD8+ T cell epitopes

The majority of the predicted epitopes were putatively restricted to a few MHC class I alleles. A structural study (molecular docking) was conducted to decipher the epitopes’ positioning in the binding groove of the MHC class I allele (HLA-A 02*01). The HLA molecule’s deposited X-ray crystal structure was retrieved from the protein data bank (PDB: 4U6Y) and dock with the various epitopes. The refined binding free and dissociation energies were determined from the docked complex.

### Population coverage analysis of CD8+ and CD4+ T cell epitopes

The selected epitopes from the HLA class I, class II families, and their respective binding leukocyte antigens were subjected to IEDB Population Coverage tool (http://tools.iedb.org/population/). This tool calculated the distribution or fraction of individuals predicted to respond to the selected epitopes with known HLA background. The IEDB tool also computes the average number of epitope hits/HLA allele combinations recognized by the entire population and the maximum and a minimum number of epitope hits recognized by 90% of the selected population. The HLA genotypic frequencies are calculated, and T cell epitopes are queried based on the area, ethnicity, and country. The entire world population was selected, followed by subcontinents and countries. Countries like Nigeria and Ghana with no deposited information on the IEDB database were included as part of the West African population.

### Designing of multi-epitope vaccine construct

Selected antigenic epitopes were scrutinized to determine which could potentially induce different Th1 and Th2 cytokines. Those with this attribute were selected for the vaccine construct. To construct a multi-epitope vaccine, we finally selected CTL, HTL, and B cell linear epitopes linked together with the help of AAY, GPGPG, and KK linkers, respectively. To boost the immunogenic profile of the selected profile epitopes, an adjuvant would be required. The outer membrane protein A (OmpA) (GenBank: AFS89615.1) was retrieved for this purpose because it serves as an agonist to the human immune receptor by interacting with antigen presenting cells **[22]**. The adjuvant was putatively added through the EAAAK linker, with the B and HTL epitopes linked together through the GPGPG linkers. These complexes were subsequently added to the CTL epitopes through the AAY linkers. The tag (6xHis-tag) was added at the C terminal end of the vaccine construct. The 6xHis-tag is one of the simplest and most widely used purification tags, with six or more consecutive histidine residues. These residues readily coordinate with transition metal ions such as Ni2+ or Co2+ immobilized on beads or resin for purification **[23]**. The vaccine peptide’s intrinsic solubility properties were conducted using the CamSol tool, which yields a solubility profile where regions (residues) with scores greater than 1 signify soluble regions. In contrast, scores lesser than -1 represents poorly soluble regions **[24]**. An overall score was generated for the entire sequence, as these amino residue scores are ranked based on their level of solubility.

### Structural modelling, assessment, and validation

All the predicted peptides 3D structures were modelled via PEPFOLD server at RPBS MOBYL portal **[25]**. PEP-FOLD is a *de novo* approach to predict peptide structures from amino acid sequences **[25–27]**. Based on structural alphabet SA letters, this method describes the conformations of four consecutive residues, couples the predicted series of SA letters to a greedy algorithm, and a coarse-grained force field **[25, 26]**. The predicted models are in cluster ranks, which are defined according to their scores. The cluster representatives correspond to the clusters’ models having the best scores, i.e., with the lowest sOPEP energy (representing the highest tm value) **[25]**. The PSIPRED v4.0 server was adopted to predict the vaccine’s secondary structure **[28]**. Simultaneously, the Swiss dock online tool was used for the tertiary structure prediction of both the vaccine construct and the human HLA class II histocompatibility antigen, DR alpha chain. To validate the generated protein structure, Procheck online tool, and Ramachandran plot analysis were generated **[29]**. The plot analysis was able to display the allowed and disallowed dihedral angles psi (ψ) and phi (ϕ) of the amino compositions, which was calculated considering the van der Waal radius of the side chain. The percentage of both the allowed and disallowed region of the glycine and proline residues plots for the modeled structure was assessed to consider if the predicted structure had a good structural precision.

### Molecular docking studies

One of the best ways to access the epitopes’ immune response is by studying their binding affinity characterizing their molecular interaction with the human HLA class I and I molecules. The binding pockets on the HLA-class I and II molecules and the human immune receptor (TRL5) was predicted using the CASTp server (http://sts.bioe.uic.edu/castp/). The CASTp server provides comprehensive and detailed quantitative characterization of topographic features of a protein. The geometric modeling principle involves the calculation strategy of alpha-shape and discrete-flow methods that are applied to the protein binding site, also the measurement of pocket-size by the program **[30]**. The protein pocket atom is identified, and then the volume and area are calculated **[31, 32]**. The program also identifies the atoms forming the rims of pocket mouth, computes how many mouth openings for each pocket, predict the area and circumference of mouth openings, finally locates cavities, and calculates their size. The secondary structures were calculated by DSSP **[30, 32]**. The HLA class I and class II allele protein’s predicted structure were utilized for molecular docking analysis with the selected epitopes (peptides) to evaluate their binding affinities. The protein structure was chemically manipulated by the expulsion of water and ligand molecules. For the peptide-protein interaction, HPEPDOCK (http://huanglab.phys.hust.edu.cn/hpepdock/) was utilized for this purpose **[33]**. It uses a hierarchical algorithm, and instead of running lengthy simulations to refine peptide conformations, HPEPDOCK also considers peptide flexibility. UCSF Chimera and Pymol tools were utilized to produce figures of docked complexes. ZDOCK server **[34]** was adopted for the molecular docking between the multiple epitope vaccine peptides and the human immune receptor (PDB: 3J0A). ZDOCK is based on the rigid-body docking program that predicts protein-protein complexes and symmetric multimers. ZDOCK achieves high predictive accuracy on protein-protein docking benchmarks, with >70% success in the top 1000 predictions for rigid-body **[35]**.

### Molecular dynamics simulation studies

The biological molecules in a peptide vaccine construct solution were studied, using the small -and wide-angle X-ray scattering (SWAXS) **[36]**. The generated curves require accurate prediction from the structural model. The predictions are complicated by scattering contributions from the hydration layer and by effects from thermal fluctuations. The MD simulations provide a realistic model for both the hydration layer and the excluded solvent, thereby avoiding any solvent-related fitting parameters while naturally accounting for thermal fluctuations **[36]**. To determine the protein compactness, the radius of gyration of the biomolecule through the Guinier analysis was also conducted. The interacting complex between the vaccine and the toll-like receptor (PDB: 3J0A) was simulated and studied based on the geometry coordinates between the superimposed protein complex. The deformability, B factor, and eigenvalues associated with the complex, including the motion stiffness was considered. Its value is directly related to the energy required to deform the structure. Deformation of the protein complex is easier at a lower eigenvalue. The covariance matrix was also considered for the simulation model. The covariance matrix indicates the coupling between the pairs of residues. It is computed using the Cα Cartesian coordinates. The elastic network of the docked complex was also computed **[36]**.

### *In Silico* codon adaptation and cloning

For the maximum expression of the vaccine in the host, a codon optimization was conducted. This process was executed using the Java Codon Adaptation Tool (JCat) to increase the translational vaccine rate in *E*. *coli* K12 system. The cleavage sites consisting of the restriction enzymes, prokaryote ribosomal binding site, and the rho-independent transcription termination left at the default state during selection. We analyzed the obtained codon adaptation index (CAI) value and GC content of the adapted sequence and assessed it within a particular threshold. Thereafter, the obtained refined nucleotide was cloned into the pET28a (+) vector prototype, utilizing the SnapGene 4.2 tool.

### Immune simulation of the chimeric peptide vaccine

The entire predicted conjugate vaccine peptide was assessed for their immunogenicity, and immune response attributes using the C-ImmSim online server (http://150.146.2.1/C-IMMSIM/index.php) **[37]**. The server uses a machine-learning basis in predicting the epitopes and the associated immune interactions. It automatically simulates three anatomical compartments, which include: (i) bone, where the hematopoietic stem cells are stimulated, and myeloid cells produced, (ii) the lymphatic organ, and (iii) the thymus where naive T cells are selected to avoid autoimmunity. We simulated the administration of three injections containing the designed peptide vaccine at an interval of four weeks i.e., days 0. 28 and 56. This prime-booster-booster approach at 4-weeks interval was employed to achieve long-lasting protective immune response based on the assessment of immune readouts from our simulation. From the default parameters, each time step was positioned at 1, 84, and 168, meaning that each time step is 8hours and time step 1 is the injection administered at time zero. So, three injections were administered at four weeks interval. However, eight injections were administered four weeks apart to stimulate repeated exposure to the antigen. In this scenario, the T cell memory will undergo continuous assessment. The Simpson index was graphically interpreted from the plot analysis **[37]**.

## Results

### Linear and discontinuous B cell epitopes

There were five promising Linear B cell epitopes with non-allergenic attributes. The peptide “VRQIAPGQTGKIAD” comparatively had the highest antigenic index than the other predicted B cell epitope candidates. A characteristic non-toxic peptide attribute makes the selected antigenic epitopes safe for vaccine design. The antigen conservancy of the epitopes across the retrieved spike protein from different geographical locations was 100% **[Table 1]**.

**Table 1:** B cells linear epitopes of SARS-CoV-2 spike glycoprotein and their immunogenic properties.

No.	Start	End	Peptide	Length	Antigenicity	Toxicity	Allergenicity
1	407	420	VRQIAPGQTGKIAD	14	1.261	Non-toxic	Non-allergen
2	18	32	LTTRTQLPPAYTNSF	15	0.79	Non-toxic	Non-allergen
3	4	18	FLVLLPLVSSQCVNL	15	0.8302	Non-toxic	Non-allergen
4	749	762	SNLLLQYGSFCTQL	14	0.800	Non-toxic	Non-allergen
5	1056	1069	PHGVVFLHVTYVPA	14	0.806	Non-toxic	Non-allergen

**NB:** All of the selected B cell are tagged B1-B5.

**Fig 1:**
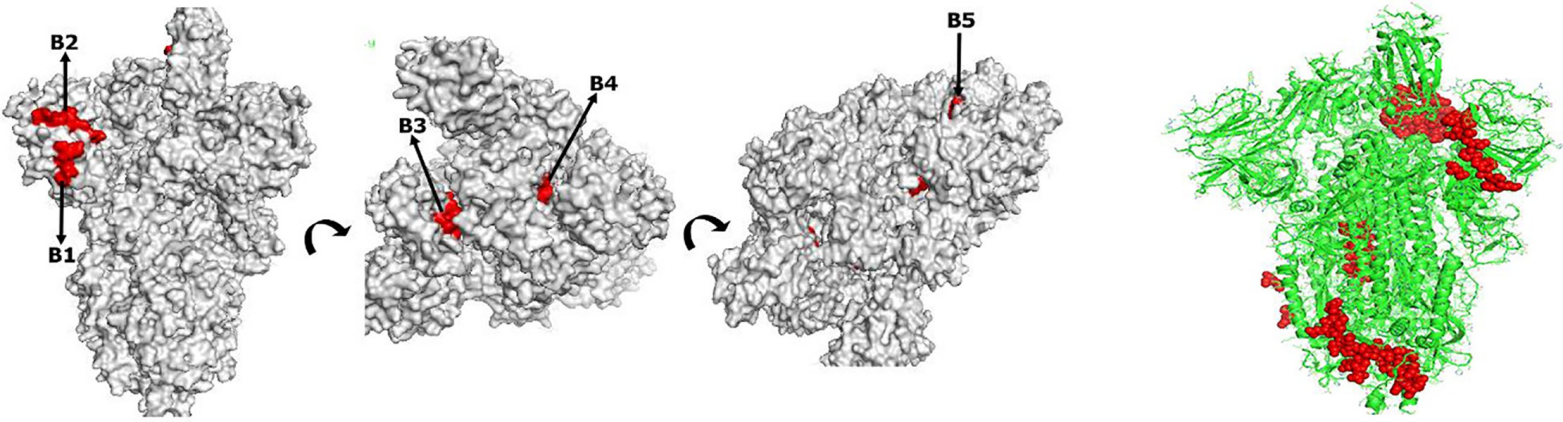
**a:** The SARS-CoV-2 crystal spike glycoprotein showing the mapped-out B cell linear epitopes. The linear epitopes are highlighted in red. Asides from the B1 and B2 epitopes that are exposed on the protein surface, the B3, B4 and B5 epitopes are embedded in the core structure, making recognition difficult. **b:** Site of the predicted discontinuous B cell epitopes overlaid on the crystal structure of SARS-CoV-2 envelope S protein. The discontinuous epitopes are shown in clusters of red spheres.

Graphically, using the Kolaskar and Tongaonkar antigenicity scale, Emini surface accessibility, and the Chou and Fasman beta-turn predictions, regions with viable immunogenic properties were determined. The scale was able to show the favorable regions across the protein that are potentially antigenic (**S1a-S1c Fig in S1 Data**). The resulting B cell linear epitopes were mapped out from the spike protein as labelled from B1 to B5 **(Fig 1A)**. The predicted discontinuous epitopes were selected from the entire protein chain component (A, B, and C) of the virus spike protein (PDB: 6VSB), and ranked based on their propensity scores. Twenty-seven discontinuous epitopes were structurally mapped out from the protein structure as shown in **Fig 1B**. Across the discontinuous epitopes of the protein chain components, the maximum contact number was ten, and the least was seven. The chain C component of the spike protein had a higher number of contact residues **[Table 2]**.

**Table 2:** Discontinuous B cell epitope contact numbers in the spike glycoprotein.

Chain ID	Residue ID	Residue Name	Contact Number	Propensity Score	Discotope score
A	809	PRO	8	2.079	-2.909
A	810	SER	7	2.066	-2.786
A	794	ILE	7	2.211	-2.745
A	793	PRO	7	2.483	-2.727
A	560	LEU	7	0.591	-2.39
A	1145	LEU	7	0.714	-1.921
A	491	PRO	6	0.255	-1.434
A	562	PHE	7	0.773	-1.289
A	1146	ASP	6	0.61	-1.017
B	1144	GLU	8	0.918	-3.082
B	811	LYS	9	1.491	-3.009
B	560	LEU	7	0.591	-2.909
B	1145	LEU	7	0.714	-2.786
B	1146	ASP	6	0.61	-2.39
B	809	PRO	8	2.079	-1.921
B	810	SER	7	2.066	-1.434
B	794	ILE	7	2.211	-1.289
B	793	PRO	7	2.483	-1.017
C	792	PRO	10	1.669	-3.331
C	1144	GLU	8	0.918	-3.082
C	811	LYS	9	1.491	-3.009
C	1145	LEU	7	0.714	-2.786
C	1146	ASP	6	0.61	-2.39
C	810	SER	8	1.996	-2.004
C	809	PRO	8	2.079	-1.921
C	794	ILE	7	2.211	-1.289
C	793	PRO	7	2.483	-1.017

Residues are shown in three-letter code, and number of contacts shows the connection of amino acid with neighboring groups

### The Cytotoxic T cell (CTL) and Helper T cell (HTL) epitopes

The spike protein sequence was scanned across multiple HLA class 1 alleles. The peptides were selected based on their percentage rankings and the number of alleles they potentially bind to. Additionally, the peptides were subjected to antigenicity test using Vaxijen 2.0. Based on the antigenicity scores, 16 epitopes were selected for the next stage of screening. The most important peptides are those with the capacity of binding with a higher number of HLA class I molecules and showing a non-allergenic attribute. Before vaccine design can be considered, the allergenicity prediction is crucial, as there is a possibility of vaccine candidates eliciting a Type II hypersensitivity reaction. Allergen 1.0 online was adopted for this analysis and the allergenicity scores show that these epitopes were non-allergenic. The non-toxicity attribute of the peptides also makes them suitable for vaccine production. Eight peptides were allergenic and eight were also non-allergenic.

The non-allergenic peptides were: GAEHVNNSY which is putatively restricted to HLA-A*01:01, HLA-B*15:01, and HLA-C*02:02, KTSVDCTMY attaches to 5 alleles: HLA-A*01:01, HLA-B*15:01, HLA-C*02:02, HLA-A*03:01, HLA-A*30:01, TTEILPVSM would be able to bind to 3 alleles: HLA-A*01:01, HLA-C*02:02, HLA-C*01:02. Other selected epitopes had similar putative restricted attachments such as “ILDITPCSF” with an antigenicity score of 1.184 and potentially attaches to seven alleles: HLA-A*01:01, HLA-C*04:01, HLA-C*02:02, HLA-C*01:02, HLA-B*15:01, HLA-A*02:01, HLA-B*13:01. The peptide GVYFASTEK would be able to bind to three alleles: HLA-A*03:01, HLA-A*30:01, HLA-C*02:02, GVYYHKNNK, ASANLAATK, VLKGVKLHY had the same similar attribute of binding to two, three and five alleles respectively **[Table 3]**.

**Table 3:** MHC-I T cell epitopes of SARS-CoV-2 spike glycoprotein.

Alleles	Start	End	Length	Peptide	Allergenicity	Antigenicity
HLA-A*01:01, HLA-B*15:01, HLA-C*02:02	652	660	9	GAEHVNNSY*	Non-allergen	0.9
HLA-A*01:01, HLA-B*15:01, HLA-C*02:02, HLA-A*03:01, HLA-A*30:01	733	741	9	KTSVDCTMY*	Non-allergen	1.1824
HLA-A*01:01, HLA-C*02:02, HLA-C*01:02	723	731	9	TTEILPVSM*	Non-allergen	1.2262
HLA-A*01:01, HLA-C*04:01, HLA-C*02:02, HLA-C*01:02, HLA-B*15:01, HLA-A*02:01, HLA-B*13:01	584	592	9	ILDITPCSF*	Non-allergen	1.184
HLA-A*01:01, HLA-B*15:01	441	449	9	LDSKVGGNY	Allergen	0.7814
HLA-A*02:01, HLA-C*02:02, HLA-C*01:02, HLA-B*13:01, HLA-C*04:01, HLA-A*30:01, HLA-B*15:01, HLA-B*08:01, HLA-B*07:02	417	425	9	KIADYNYKL	Allergen	1.664
HLA-A*02:01, HLA-C*02:02, HLA-C*01:02, HLA-A*30:01, HLA-B*08:01, HLA-C*04:01, HLA-B*13:01	1060	1068	9	VVFLHVTYV	Allergen	1.512
HLA-A*02:01, HLA-B*08:01, HLA-C*04:01, HLA-C*01:02, HLA-C*02:02, HLA-A*01:01, HLA-B*13:01, HLA-B*07:02	109	117	9	TLDSKTQSL	Allergen	1.0685
HLA-A*03:01, HLA-A*30:01, HLA-C*02:02	89	97	9	GVYFASTEK*	Non-allergen	0.7112
HLA-A*03:01, HLA-A*30:01	142	150	9	GVYYHKNNK*	Non-allergen	0.8264
HLA-A*03:01, HLA-A*30:01, HLA-C*02:02	1020	1028	9	ASANLAATK*	Non-allergen	0.7014
HLA-A*03:01, HLA-A*30:01	409	417	9	QIAPGQTGK	Allergen	1.8297
HLA-A*03:01, HLA-A*30:01, HLA-B*15:01, HLA-C*02:02, HLA-A*01:01	1264	1272	9	VLKGVKLHY*	Non-allergen	1.2378
HLA-A*03:01, HLA-A*30:01, HLA-C*02:02	349	357	9	SVYAWNRKR	Allergen	0.765
HLA-A*03:01, HLA-A*30:01	725	733	9	EILPVSMTK	Allergen	1.6842
HLA-A*03:01, HLA-A*30:01	378	386	9	KCYGVSPTK	Allergen	1.4199

* The selected epitopes

For the HLA class II T cells epitopes, the spike protein sequence was also scanned through a large number of the MHC-II alleles. Twelve epitopes were selected based on their antigenic properties. All of these selected non-allergenic epitopes are capable of eliciting an immune response by inducing either or all of IFN-γ, IL-4 and IL-10 cytokines. The peptide “GYFKIYSKHTPINLV” was the only candidate to induce all of the three cytokines, which was intriguing. Selection of these epitopes was also centered on their putative bindings to a large number of MHC-II alleles. The peptide “FAMQMAYRF”, with an antigenicity score of 1.0278, attaches to 8 HLA alleles: -DRB1*01:02, -DRB1*01:04, -DRB1*01:03, -DRB1*01:01, -DRB1*01:05, -DRB1*07:01, -DRB1*04:01, and -DRB1*03:01. The epitope “FRVQPTESI”, with an antigenicity score of 0.9396, is also restricted to 8 HLA alleles: -DRB1*04:01, -DRB1*01:01, -DRB1*01:05, -DRB1*07:01, -DRB1*01:03, -DRB1*01:02, -DRB1*03:01, and -DRB1*01:04. The HLA-DRB1 is the most common and versatile MHC-II molecule. The entire putative attachments of the selected epitopes are summarized **[Table 4]**. The conservancy level of the epitopes across the retrieved spike protein sequences from different geographical location was 100%.

**Table 4:** MHC class II T-cell epitopes of SARS-CoV-2 spike glycoprotein.

S/N	Core peptides	Peptides	Start	End	Length	Allergenicity	IFN-γ	IL-4	IL-10	MHC II Alleles	Antigenicity
1.	FAMQMAYRF	IPFAMQMAYRFNGIG	896	910	15	Non-allergenic	-	+	-	DRB1*01:02, DRB1*01:04, DRB1*01:03, DRB1*01:01, DRB1*01:05, DRB1*07:01, DRB1*04:01, DRB1*03:01	1.0278
2.	FRVQPTESI	IYQTSNFRVQPTESI	312	326	15	Non-allergenic	+	+	-	DRB1*04:01, DRB1*01:01, DRB1*01:05, DRB1*07:01, DRB1*01:03, DRB1*01:02, DRB1*03:01, DRB1*01:04	0.9396
3.	FQTRAGCLI	STGSNVFQTRAGCLI	637	651	15	Non-allergenic	+	-	+	DRB1*01:03	1.7332
4.	CVLGQSKRV	KMSECVLGQSKRVDF	1028	1042	15	Non-allergenic	-	+	-	DRB1*01:03	0.9083
5.	FLHVTYVPA	VVFLHVTYVPAQEKN	1060	1074	15	Non-allergenic	+	-	+	DRB1*04:01, DRB1*07:01, DRB1*01:01, DRB1*01:05	1.3346
6.	LQIPFAMQM	ALQIPFAMQMAYRFN	893	907	15	Non-allergenic	-	+	-	DRB1*01:02, DRB1*01:04, DRB1*01:03, DRB1*01:01, DRB1*01:05	1.0680
7.	IGINITRFQ	IGINITRFQTLLALH	231	245	15	Non-allergenic	-	-	+	DRB1*03:01	1.3386
8.	VFQTRAGCL	NVFQTRAGCLIGAEH	641	655	15	Non-allergenic	-	-	+	DRB1*07:01, DRB1*01:01, DRB1*01:05	1.7094
9.	FTISVTTEI	TNFTISVTTEILPVS	716	730	15	Non-allergenic	-	+	+	DRB1*07:01, DRB1*04:01, DRB1*01:05, DRB1*01:01	0.8535
10.	YFKIYSKHT	GYFKIYSKHTPINLV	199	212	15	Non-allergenic	+	+	+	DRB1*01:03, DRB1*01:02, DRB1*04:01, DRB1*01:01, DRB1*01:05, DRB1*07:01, DRB1*01:04	0.9056
12.	AALQIPFAM	AALQIPFAMQMAYRF	892	906	15	Non-allergenic	-	+	-	DRB1*01:02	0.7747
13.	VLSFELLHA	RVVVLSFELLHAPAT	509	523	15	Non-allergenic	+	+	-	DRB1*01:02	1.0776

+ Induced,—non-induced. Antigenicity threshold at 0.7

### Population coverage of the CTL and HTL epitopes

HLA allelic distribution differs among diverse geographical regions and ethnic groups around the globe. It is therefore imperative to consider the population coverage in designing a viable epitope-based vaccine relevant for global populations. The selected CD8^+^ T cell epitopes exhibited a higher individual percentage cover when queried with the entire world population. The HLA hits across the entire population revealed that approximately 81% of the world individuals are capable of responding to a median of three CTL epitopes **[Table 5]**.

**Table 5:** Coverage of individual epitope (MHC class I) in the world.

Epitope	Coverage	HLA allele									Total HLA hits
		(Genotypic frequency (%))									
	Class I	HLA-A*01:01	HLA-A*02:01	HLA-A*03:01	HLA-A*30:01	HLA-B*13:01	HLA-B*15:01	HLA-C*01:02	HLA-C*02:02	HLA-C*04:01	
		(10.09)	(24.39)	(9.77)	(2.18)	(1.57)	(5.65)	(6.1)	(5.52)	(11.93)	
ILDITPCSF	73.40%	**+**	**+**	**-**	**-**	**+**	**+**	**+**	**+**	**+**	**7**
KTSVDCTMY	46.77%	**+**	**-**	**+**	**+**	**-**	**+**	**-**	**+**	**-**	**5**
VLKGVKLHY	46.77%	**+**	**-**	**+**	**+**	**-**	**+**	**-**	**+**	**-**	**5**
TTEILPVSM	33.48%	**+**	**-**	**-**	**-**	**-**	**-**	**+**	**+**	**-**	**3**
GAEHVNNSY	31.54%	**+**	**-**	**-**	**-**	**-**	**+**	**-**	**+**	**-**	**3**
GVYFASTEK	27.95%	**-**	**-**	**+**	**+**	**-**	**-**	**-**	**+**	**-**	**3**
ASANLAATK	27.95%	**-**	**-**	**+**	**+**	**-**	**-**	**-**	**+**	**-**	**3**
GVYYHKNNK	20.35%	**-**	**-**	**+**	**+**	**-**	**-**	**-**	**-**	**-**	**2**
**Epitope set**	**81.05%**	**5**	**1**	**5**	**5**	**1**	**4**	**2**	**7**	**1**	**31**

+: restricted

-: not restricted

shaded column: genotypic frequency of this allele is 0 (zero)

However, the population coverage for the CD4^+^ T cell epitopes was comparatively lower compared to the CD8^+^ T cell epitopes, with average population coverage of 55.23% and recognition of a median of 2 epitopes **[Table 6]**.

**Table 6:** Coverage of individual epitope (MHC class II) in the world.

Epitope	Coverage	HLA allele								Total HLA hits
		(Genotypic frequency (%))								
	Class II	HLA-DRB1*01:01	HLA-DRB1*01:02	HLA-DRB1*01:03	HLA-DRB1*01:04	HLA-DRB1*01:05	HLA-DRB1*03:01	HLA-DRB1*04:01	HLA-DRB1*07:01	
		(6.65)	(1.8)	(0.92)	(0.01)	(0)	(10.47)	(6.46)	(10.71)	
IPFAMQMAYRFNGIG	55.23%	**+**	**+**	**+**	**+**	**+**	**+**	**+**	**+**	**8**
IYQTSNFRVQPTESI	55.23%	**+**	**+**	**+**	**+**	**+**	**+**	**+**	**+**	**8**
GYFKIYSKHTPINLV	41.83%	**+**	**+**	**+**	**+**	**+**	**-**	**+**	**+**	**7**
VVFLHVTYVPAQEKN	38.05%	**+**	**-**	**-**	**-**	**+**	**-**	**+**	**+**	**4**
TNFTISVTTEILPVS	38.05%	**+**	**-**	**-**	**-**	**+**	**-**	**+**	**+**	**4**
NVFQTRAGCLIGAEH	28.63%	**+**	**-**	**-**	**-**	**+**	**-**	**-**	**+**	**3**
IGINITRFQTLLALH	17.84%	**-**	**-**	**-**	**-**	**-**	**+**	**-**	**-**	**1**
ALQIPFAMQMAYRFN	16.07%	**+**	**+**	**+**	**+**	**+**	**-**	**-**	**-**	**5**
AALQIPFAMQMAYRF	3.19%	**-**	**+**	**-**	**-**	**-**	**-**	**-**	**-**	**1**
RVVVLSFELLHAPAT	3.19%	**-**	**+**	**-**	**-**	**-**	**-**	**-**	**-**	**1**
KMSECVLGQSKRVDF	1.64%	**-**	**-**	**+**	**-**	**-**	**-**	**-**	**-**	**1**
STGSNVFQTRAGCLI	1.64%	**-**	**-**	**+**	**-**	**-**	**-**	**-**	**-**	**1**
**Epitope set**	**55.23%**	**8**	**6**	**6**	**4**	**8**	**3**	**6**	**7**	**48**

+: restricted

-: not restricted

the genotypic frequency of this allele is 0 (zero)

**Fig 2:**
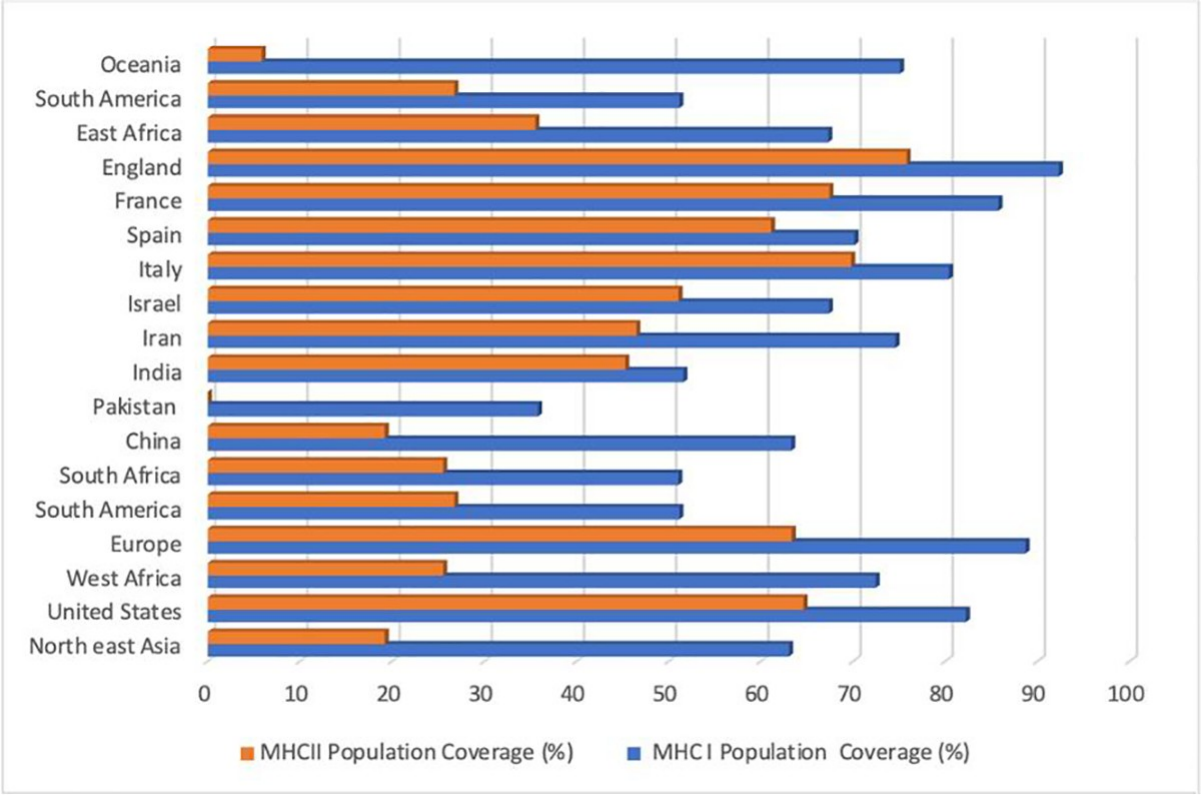
Selected continents and countries’ coverage of the MHC class I and II T cell epitopes.

Based on the selection of the continents and countries, the European populace would likely show a significant response to the selection of putative HLA class I restricted epitopes. England, France, United States, Italy, and Oceania had the highest population coverage of 92.31%, 85.75%, 82.22%, 80.39%, and 75.07% respectively, while the Pakistan population had the lowest population coverage at 35.8%. The population cover for the MHC class II epitopes in contrast to the MHC class I epitopes was considerably lower. The striking observation was 0% coverage exhibited by the Pakistan population **[Fig 2]**.

### Binding orientations of the CTL and HTL epitopes in HLA-A*02:01 and HLA-DRB1*01:01 groove

**Fig 3:**
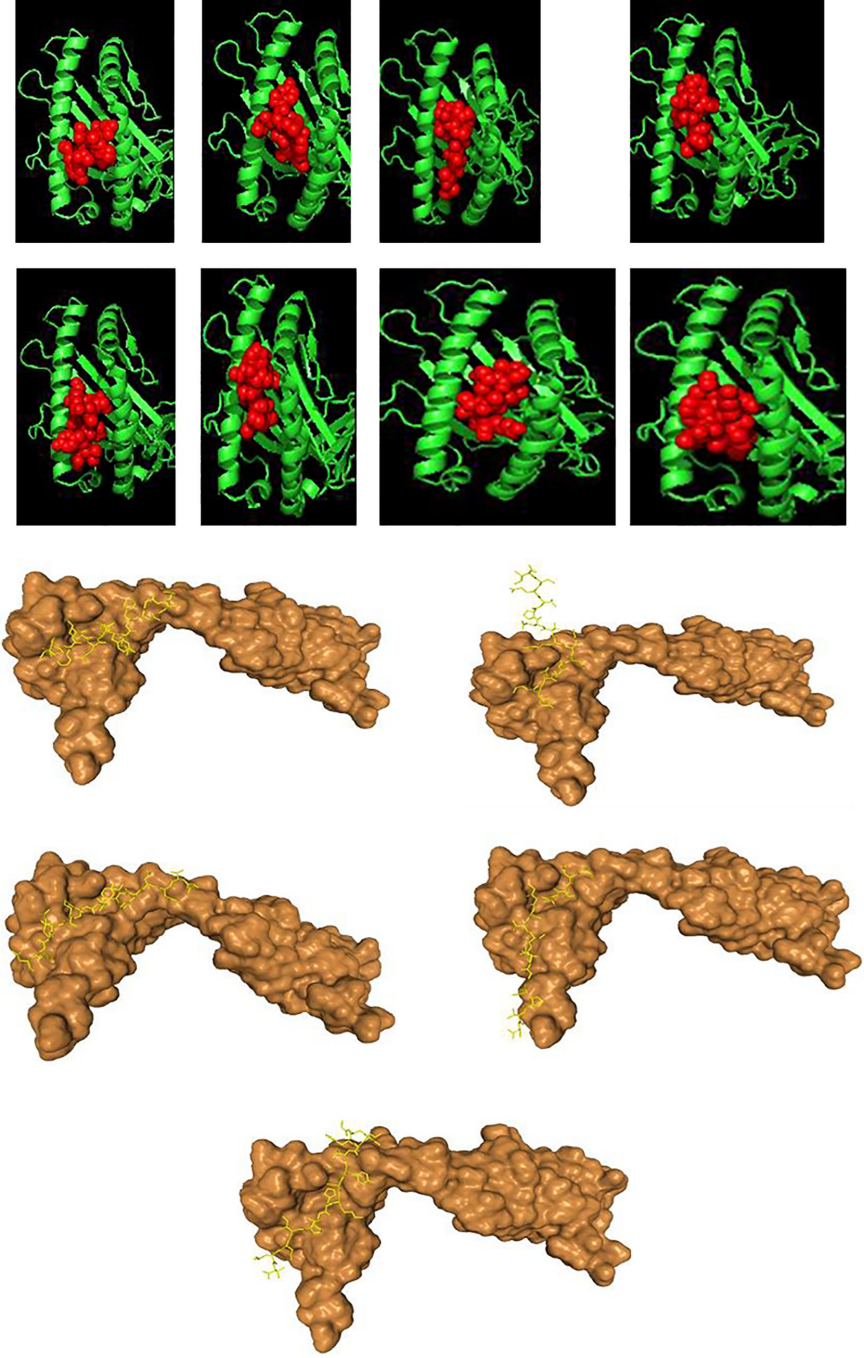
**(a-h)**: Differential binding of the CTL epitopes with HLA*01:01. The MHC protein is displayed in green color and the epitopes are highlighted in red-clustered balls. The epitopes are listed according as follows. (**a**) GAEHVNNSY, (**b**) KTSVDCTMY, (**c**) TTEILPVSM, (**d**) ILDITPCSF, (**e**) GVYFASTEK, (**f**) GVYYHKNNK, (**g**) ASANLAATK, and (**h**) VLKGVKLHY. **(I-m):** Differential binding of the HTL epitopes with HLA-DRB1*01:01. The MHC protein is displayed in surface brown and the epitopes are highlighted in yellow licorice. The HTL epitopes are listed according as follows. (I) IPFAMQMAYRFNGIG, (**j**) IYQTSNFRVQPTESI, (**k**) GYFKIYSKHTPINLV, (**l**) VVFLHVTYVPAQEKN, and (**m**) TNFTISVTTEILPVS.

The selected CTL and HTL antigenic epitopes were docked individually with the alleles they were highly restricted to, which was HLA-A*02:01 for the CTL epitopes and HLA-DRB1*01 for the HTL epitopes. The differential binding patterns of the CTL epitopes were examined **[Fig 3A–3H]**.

Major class histocompatibility class II amino acid sequences are highly polymorphic within a population, and correlate with individual differences in response to infectious agents and vaccines. It is therefore imperative to structurally examine how the CD4+ epitopes recognize peptide fragments of antigens that lie in the antigen groove of the MHC-II protein. The protein structure of the retrieved human HLA class II histocompatibility antigen, DRB1 beta chain (human leukocyte antigen DRB1, HLA- DRB1*01:01) (PDB: 1AQD) was retrieved for the molecular docking of the HTL epitopes because it was the most occurring allele that the peptides were restricted to. The HTL epitopes with good population cover were chosen for molecular docking with HLA-DRB1*01:01.

The peptides: IPFAMQMAYRFNGIG, IYQTSNFRVQPTESI, VVFLHVTYVPAQEKN, TNTTISVTTEILPVS, and GYFKIYSKHTPINLV, were the selected epitopes. The binding free energy characterizing the HLA-DRB1 antigenic binding groove and the interacting epitopes alongside the corresponding amino residues were evaluated. The epitopes exhibited different binding pattern with the MHC class II groove. Few of the binding peptides had a flanking region outside the groove. Amino acids that are outside of the “core” peptide region extends out of the open MHC-II binding groove forming the peptide flanking regions at both the N- and C-terminus **[Fig 3I–3M]**.

The epitope “IYQTSNFRVQPTESI” had the most extensive flanking non-binding region with some of part of the peptide protruding completely out of the groove. IPFAMQMAYRFNGIG had the best binding free energy score, with TNTTISVTTEILPVS with the least binding energy **[Table 7]**.

**Table 7:** Docking properties of HLA-DRB1*01:01 restricted epitopes.

	Epitopes	Binding free energy (kcal/mol)	Interacting residues of the MHC II protein
a.	IPFAMQMAYRFNGIG	-18.92	PHE 15, GLN 146, PHE 152, PHE 14, VAL 88
b.	IYQTSNFRVQPTESI	-26.59	TRP 58, TRP 6, LEU 8, LEU 64, TYP 57
c.	VVFLHVTYVPAQEKN	-28.34	ARG 45, LEU 24, ARG 36, VAL 41, VAL 47
d.	TNTTISVTTEILPVS	-30.89	SER 123, LYS 9, VAL 126, HIS 13, LEU 144
e.	GYFKIYSKHTPINLV	-14.48	ILE 124, LEU 144, VAL 139, ALA 137, SER 141

### Construction of the peptide vaccine

**Fig 4:**
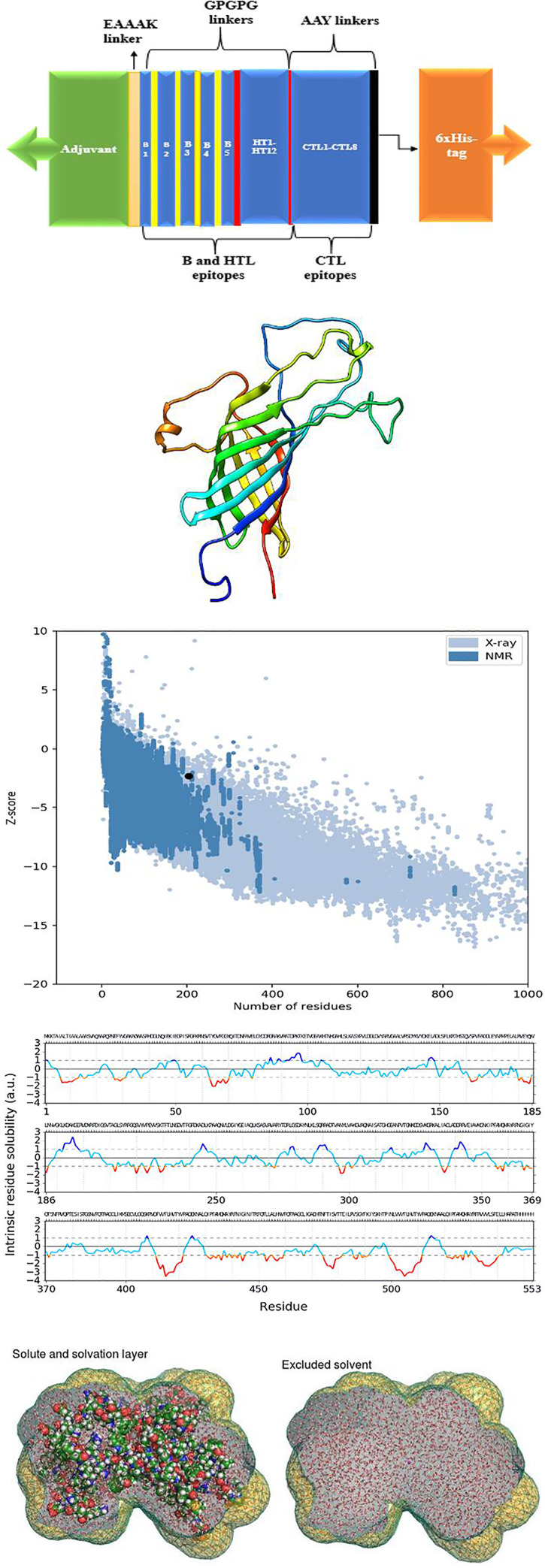
(**a**) Schematic presentation of the vaccine containing an adjuvant (green) linked with the multi-epitope sequence through an EAAAK linker. The B and HTL epitopes are linked together via the GPGPG linkers while the CTL epitopes are linked with the help of AAY linkers. The 6x-His tag at the carboxyl end. (**b**) Tertiary structure of the vaccine. (**c**) Validation of the structure with a Z score of −2.32. (**d**) Intrinsic solubility profile. Residues lesser than -1 depicts the hydrophobic core of the vaccine peptide. (**e**) The solute and solvation layer of the vaccine.

The multiple epitope peptide vaccine consists of 553 amino residues from 25 selected antigenic B and T cells epitopes, covalently linked with an immuno-adjuvant **[Fig 4A]**. The tertiary structure of the multiple epitope vaccine was also obtained **[Fig 4B]**, and the structural validation was assessed using ProSA-web which predicts the overall quality of the model indicated in the form of z-score. If the z-scores of the predicted model are outside the range of the characteristic for native proteins, it indicates the erroneous structure. The Z-score was -2.32 for the vaccine predicted model indicating a relatively good model **[Fig 4C]**. Before the addition of the OmpA protein adjuvant, the conjugated vaccine was highly antigenic with a score of 0.8 after subjecting it to Vaxijen server, signifying that the vaccine is viable at inducing cellular and humoral immune response without the aid of an adjuvant. However, an adjuvant was added to further boost the immunogenic properties to 0.85. A structural appraisal of the secondary structure of the vaccine revealed 14% alpha helix, 41% beta strand and the disordered region was 17%.

### Physiochemical, solubility and solvation properties of the vaccine

The physicochemical parameters and solubility properties of a vaccine candidates help to define the efficacy and effectiveness of the vaccine. The molecular weight of the vaccine was 60728.51 Da and the bio-computed theoretical pI was 9.30, with an estimated half-life of 30 hours. The instability index was 27.84, signifying that the vaccine is stable in a solvent environment (>40 signifies instability). The aliphatic index is computed to be 88.21, with a GRAVY score of -0.056. The intrinsic vaccine solubility at a neutral pH 7 revealed the hydrophilic and hydrophobic core of the vaccine construct **[Fig 4D]**. The overall solubility value of the vaccine was -2.632908 signifying hydrophilic property. The stability of the vaccine construct was assessed considering the radius of gyration and solvent density. The solvent density of the vaccine is 334 e/nm^-3^, the envelope distance is 7 Å, number of q values is 101 and the heavy atoms is a total of 1589. The protein contains 3143 solutes atoms and 14365 water molecules. The solute has zero charges with the distance of envelope from the solute at 0.7nm while the maximum diameter of the solute is 7.3673 nm **[Fig 4E]**.

### Molecular docking between the adjuvant linker of the vaccine and the toll-like receptor (TLR5)

**Fig 5:**
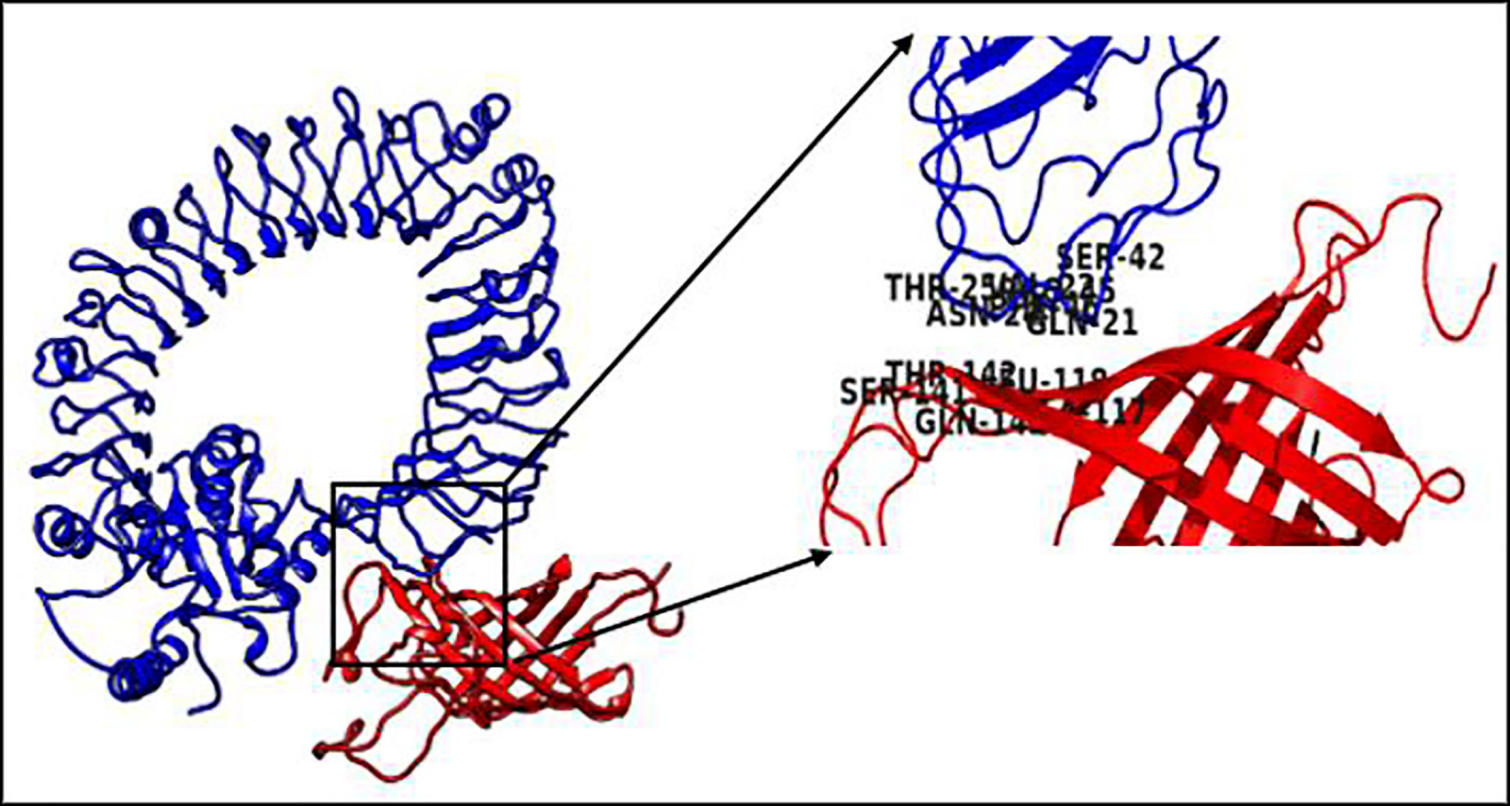
The molecular interaction of the vaccine and TLR5 receptor. The vaccine chain is highlighted in red and the toll like receptor in blue.

The TLR5 was selected due to its immunomodulatory ability to trigger IFN-g as well as activation of type I IFN responses **[38]**. This was attested in the study, as our selected CD4+ epitopes elicited both the Th1 and Th2 cytokines. The molecular interaction between the vaccine and the TLR5 (PDB: 3J0A), was evaluated considering their refined binding energies and various interacting residues. Based on our structural docking analysis, the conformational triggering of the TLR5 receptor was influenced by the adjuvant linker and not the conjugated epitopes. Structurally, the adjuvant linker binds to the A chain monomer of the toll-like receptor. The interface amino residues of the receptor were: PRO 20, GLN 21, VAL 22, LEU 23, ASN 24, THR 25, PRO 45, and PHE 46 respectively from the A chain, forming a hydrogen bond with the interacting adjuvant residues: ASN 91, GLN 143, HIS 89, ALA 117, LEU 118, VAL 119, ARG 120, THR 142 and SER 141 **[Fig 5]**.

The binding energy (ΔG) and dissociation constant (K_d_) predicted values of the protein-protein complex were -12.2 kcal mol^-1^ and 1.0E-09 at 25.0°C respectively.

### Molecular dynamics simulations

**Fig 6:**
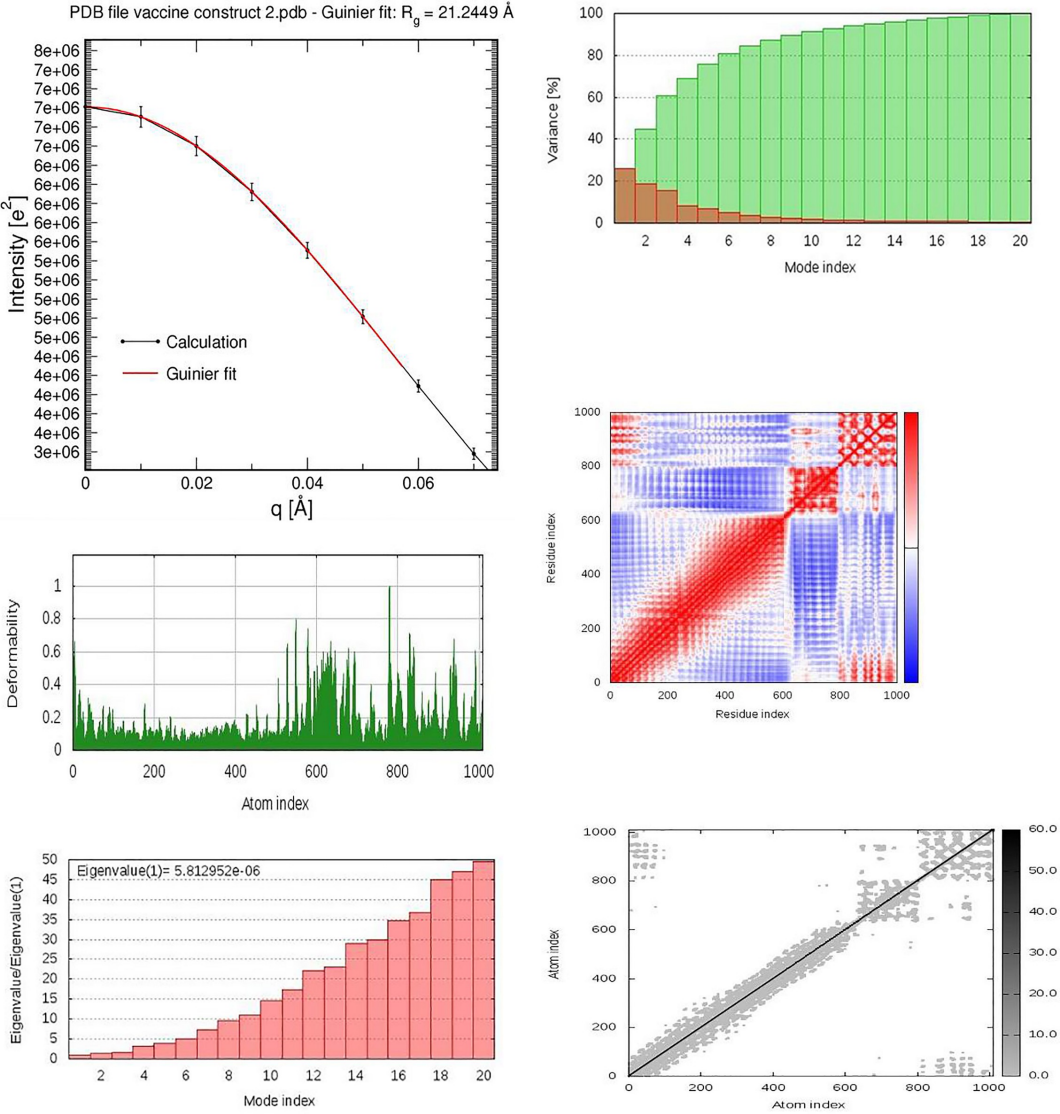
**a:** The radius of gyration of the peptide vaccine. **(b-f)**: Molecular dynamics simulation of the vaccine-TLR5 complex, showing (**b**) eigenvalue; (**c**) deformability; (**d**) B-factor; (**e**) Covariance matrix indicates coupling between pairs of residues (red), uncorrelated (white) or anti-correlated (blue) motions and (**f**) elastic network analysis which defines which pairs of atoms are connected by springs.

The rigidity of the peptide vaccine system was examined by evaluating the radius of gyration (Rg) values. The analyzed data shows that the average Rg value was 21.0067 nm, indicating that the protein system retained its stability throughout the 85.5 ns time span of the MD simulation **[Fig 6A]**, signifying the relative peptide stability. The molecular interaction between the vaccine peptide and the TLR5 was screened for their protein stability, B factor mobility, and deformity. This analysis relies on the associated coordinates of the docked protein complex **[Fig 6B–6F]**.

The eigenvalue found for the superimposed complex was 5.812952e-06. The low eigenvalue for the complex signifies easier deformation of the complex, indicating that the docking analysis between the vaccine and the TLR5 will activate immune cascades for destroying the antigens.

### Codon optimization and *In silico* cloning

**Fig 7:**
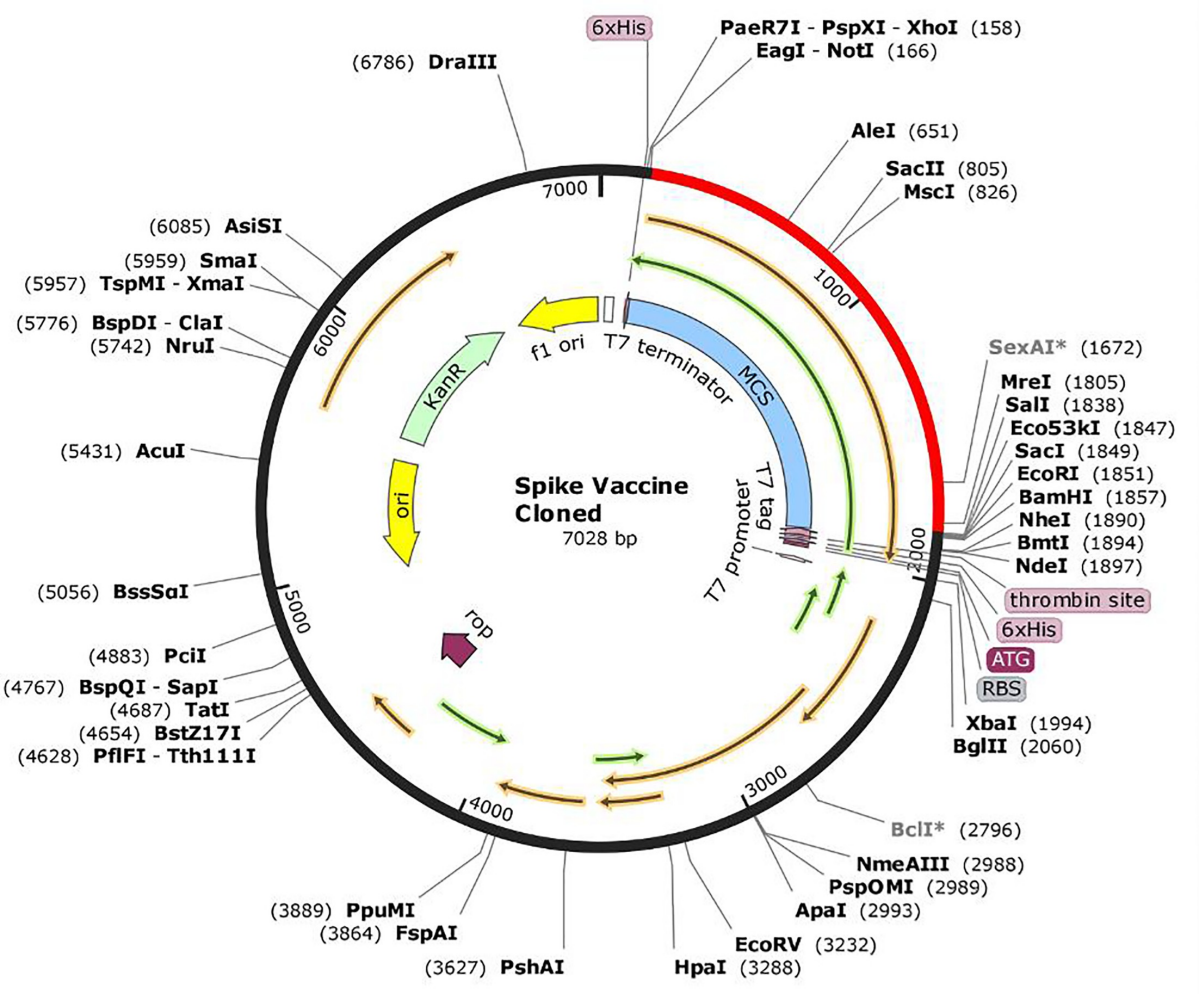
*In silico* cloning of the final vaccine construct into pET28a (+) expression vector where the red part indicates the coding gene for the vaccine surrounded between EagI-NotI (166) and SAlI (1838) while the vector backbone has shown in a black circle. MCS represents the multiple cloning sites.

The length of the optimized vaccine codon sequence was 1659 nucleotides. The GC content of the cDNA sequence and codon adaptive index was calculated as 50.8%, which still falls within the recommended range of 30–70%, for effective translational efficiency. The calculated codon adaptive index was 0.93, falling within the range of 0.8–1.0, signifying the effective expression of the vaccine constructs in the *E*. *coli*. EagI-NotI and SAlI sites were subsequently cloned into the pET28a (+) vector. The estimated length of the clone was 7.028 kbp **[Fig 7]**.

### Immune simulation of the chimeric peptide vaccine

**Fig 8:**
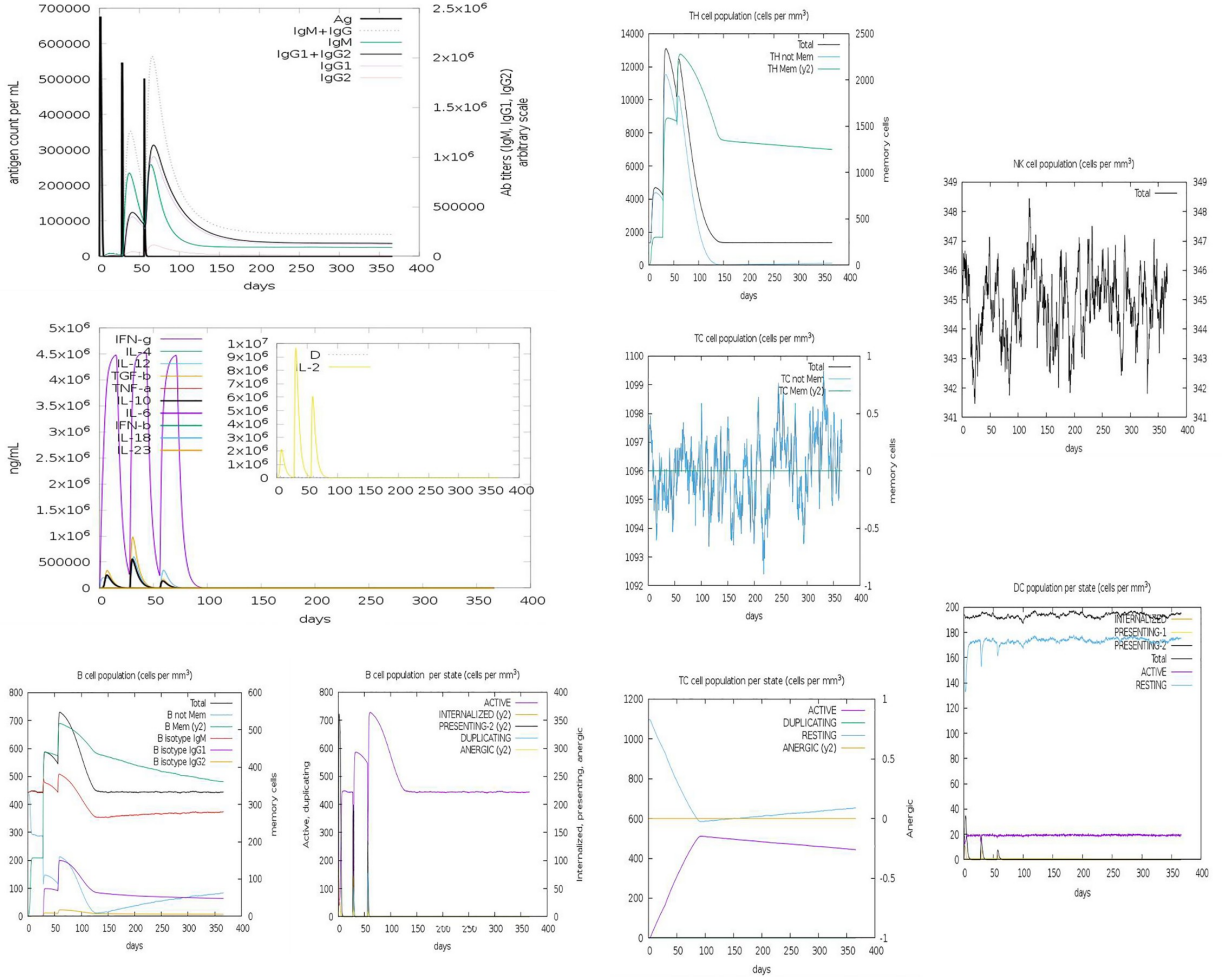
The induced immune cells by the peptide vaccines. (**a**) The concomitant decrease in antigen level with rise in immunoglobin activities. (**b**) Induced array of cytokines during prime boost. (**c)** B lymphocytes: total count, memory cells, and sub-divided in isotypes IgM, IgG1 and IgG2. (**d)** CD4 T-helper lymphocytes count. (**e)** CD4 T-helper lymphocytes count. (**f)** CD8 T-cytotoxic lymphocytes count. (**g)** CD8 T-cytotoxic lymphocytes count per entity-state. (**h)** Natural Killer cells (total count). (**i)** Dendritic cells.

At every administration and repetitive exposure to the attenuated peptide vaccine, there was a significant increase in the antibody response with a simultaneous decrease in the antigen level. There was a predominantly IgM > IgG humoral response indicating some levels of seroconversion. Although humoral response peaked higher to the booster than the prime dose, IgM was still higher than IgG response (IgG was higher to first booster than prime dose) which shows some level of seroconversion **[Fig 8A]**. Also, following the prime dose, there was an initial spike in IFN-g response (associated with both CD8+ T-cell and CD4+ Th 1 response), and also IL-10 & TGF-b cytokines response associated with T-reg phenotype **[Fig 8B]**. These immune readouts also associate with a wider base in the spike peak of antigenic quantification. However, following the first booster dose, IFN-g also peaked. Given the second booster dose on day 56, IgG response was faster and higher than IgM response (which shows full seroconversion and B-cell memory response), faster antigen clearance as shown by a narrow base of the antigenic spike compared to previous doses, and a lower T-reg associated cytokines (TGF-b & IL-10) compared to first booster dose response. Overall, results showed with each vaccination schedule revealed that there was concomitant increase in immune response. Analysis of B-cell population per cell showed overall B-cell population and B-cell memory responses were higher and stable showing minimal decay for over 350 days **[Fig 8C]**. The same results go for B-cell population per state where active B-cell population was higher and stable for the same number of periods **[Fig 8D]**. Following the prime dose, there was a concomitant rise in Th effector cell phenotype, and lower Th-memory readout **[Fig 8E]**. There was also a corresponding higher Th effector and Th memory phenotype (although Th eff >Th memory) **[Fig 8F]**. in **Fig 8G**, it shows T-cell response per state, where active T-cell population from around day 50 increased and remained steady till around day 350. Also, there was concomitant increase in dendritic cells and natural killer cells activities **[Fig 8H–8I]** throughout the duration of the simulation. This points to another good performance indicator of the vaccine construct showing its ability to stimulate the right immunological compartment for effective response.

This points to another good performance indicator of the vaccine construct showing its ability to stimulate the right immunological compartment for effective response.

## Discussion

The emergence of new coronavirus strain SARS-CoV-2 viral diseases is a global threat, responsible for the death of many across the globe, including health care workers **[1]**. Therefore, there is an urgent need for therapeutics and preventive measures that could confer protection against this enigma. Our study was therefore centered on using an epitope peptide-based vaccine design against the SARS-CoV-2 spike protein complex. We successfully developed a peptide vaccine after a rigorous round of *in silico* screenings and conditions in selecting the epitopes, using an array of immuno-informatics tools. Criteria such as the elicitation of immune response with lesser or no potential infectious abilities were considered before each epitope selection, using stipulated thresholds. Both the B cell and T cell epitopes were predicted in this study. B cells recognize solvent-exposed antigens through antigen receptors, named as B cell receptors (BCR), consisting of membrane-bound immunoglobulins **[39]**. The B cell epitopes were selected based on surface accessibility, and Kolaskar and Tongaonkar antigenicity scale methods. Five antigenic B cell epitopes were predicted in the study. The peptide also has a non-toxicity attribute, making it a safe vaccine candidate.

The T cell antigenic epitopes capable of binding a large number of MHC I and MHC II alleles were predicted using various tools, and selections were made based on the recommended thresholds. T cell epitopes presented by MHC class I molecules are typically peptides between 8 and 11 amino acids in length, whereas MHC class II molecules present longer peptides, 13–17 amino acids in length **[40, 41]**. The CD8^+^ T cell recognizes the antigen of a pathogen after its attachment with the MHC I molecules, therefore triggering a cytotoxic response against the pathogen **[42–45]**. Eight promising CD8^+^ T cell epitopes were predicted. These peptides could be capable of eliciting a cytotoxic response with their respective antigenic properties.

Studies have shown that specific antibodies are produced against SARS-CoV and these antibodies were persistent in patients who recovered from the SARS infection **[46]**. This similar scenario occurs with the Middle East respiratory syndrome (MERS)-CoV antibodies that can only identify some of the patients who had MERS-CoV infections, and these titers substantially plummet within the first 6 months of infection **[47]**. The SARS-CoV-2, just like the SARS-COV and MERS-CoV belongs to the beta-coronavirus genus of the Corornaviridae family. A study by Ni et al. reported that specific antibodies produced against SARS-COv-2 were disappeared in a convalescent COVID-19 patient within 3 months **[48]**. This could suggest that the specific antibodies produced against SARs-CoV-2 are short-lived in this convalescent COVID-19 patient and might not neutralize SARS-CoV-2 infection.

Prediction of peptide binding to MHC II molecules readily discriminate CD4 T-cell epitopes, but cannot tell their ability to activate the response of specific CD4 T-cell subsets (e.g., Th1, Th2, and Treg). However, there is evidence that some CD4 T-cell epitopes appear to stimulate specific subsets of Th cells **[42, 43]**. The ability to distinguish the epitopes capable of inducing distinct responses is highly imperative in vaccine development. Readouts from our immune simulations shows that both Th1 and Th2 responses can be stimulated our vaccine construct inferring from the correlating cytokine responses when subjected to in vivo conditions. The latter condition remains to be validated.

However, it is evident that our vaccine design has numerous advantages as multi epitopic conjugate, such that it can induce both protective cellular and humoral immunity in a long-term basis (up to 360 days), provided 3 doses of the vaccine were administered.

The docking scores involving the predicted epitopes and the MHC II molecules were comparatively evaluated. Considering the docking attributes of the MHC II epitopes, they displayed a varied binding putative attribute. After our structural examination, some of the epitopes’ core peptides had a flanking region that is away from the MHC class II binding groove. Generally, MHC-II peptides contain a central binding motif of nine core amino residues that specifically attach to the MHC II binding groove. These core peptides interact with the allelic specific pockets of the MHCII binding groove **[49–51]**.

Considering the population cover for the MHC I and II epitopes, the European population significantly show a potential response to the selected epitopes, however, we suggest that the IEDB population coverage tool have less MHC class I and II deposition of alleles from continents like Africa and Asia, compared to the European and Americans. Immunization of the MHCII T cell epitopes will confer protection to 80.88% of the world population, while MHCII T cell epitopes will confer protection to 55.23%. It is imperative to know that specific interactions with high binding affinity epitope / HLA allele class II molecule unleash protective and specific adaptive immune response **[39–42]**.

The designed vaccine construct was predicted to be stable, soluble (i.e., hydrophilic) and with increased thermostability, as depicted in its physicochemical characteristics. The molecular weight of the vaccine and its high pI value signifies the efficacy as well as the stability of the vaccine construct since proteins having <110kD molecular weight are considered good vaccine candidates **[43, 44]**. Apart from size, surface properties like surface charge and hydrophobicity can affect a designed vaccine candidate. Neutral or negatively charged molecules are preferred and a balance between its hydrophobicity and hydrophilicity is crucial in designing vaccine candidates **[45]**.

## Conclusion

This is a novel approach to predicting SARS-CoV-2 epitope peptide-based vaccine targeting the spike protein, utilizing immune-informatics tools and immune simulation measures. These predicted antigenic epitopes would hasten the production of protective vaccine for patients around the world whose immune system has been compromised. It was intriguing that our vaccine was able to stimulate neutralizing antibody as well as cellular response components well up to 350 days after the last second booster shot as computationally derived. This corresponding decrease in antigen level with each simulated vaccination suggests that the immune response may be capable of swift clearance when tested under *in vivo* conditions. Our selected epitopes (B and T cells) will constitute a good vaccine candidate against the spike protein. In future, other effective stimulants or adjuvants that could facilitate the rapid response of cells to antigens will be considered and assessed.

### Limitation of the study

This study employed solely an in-silico approach in the design of the multiepitope peptide vaccine construct and validation of the simulated vaccinations with the immune responses. Therefore, although the study was performed with stringent standards to ensure success and translation under-*in vivo* conditions, there might be mild deviations in results obtained with animal models as simulations were done with human parameters and available population studies.

## Supporting information

S1 Data (DOCX)
