# Supplementary material for: Immuno-informatics design of a multimeric epitope peptide based vaccine targeting SARS-CoV-2 spike glycoprotein
Source: PLoS One. 2021 Mar 17;16(3):e0248061. doi: 10.1371/journal.pone.0248061 (PMC7968690; doi:10.1371/journal.pone.0248061)
Supplement: S1 Data — (DOCX) [file pone.0248061.s001.docx]

**S1 Data.**


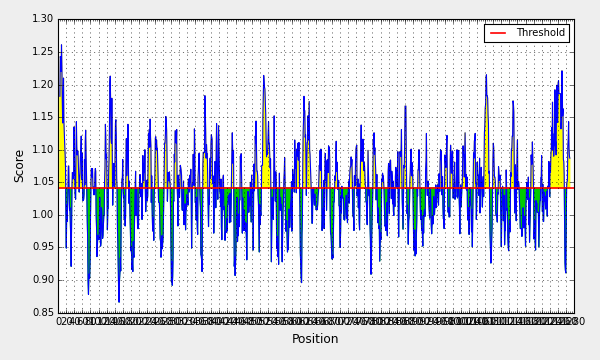


**a**


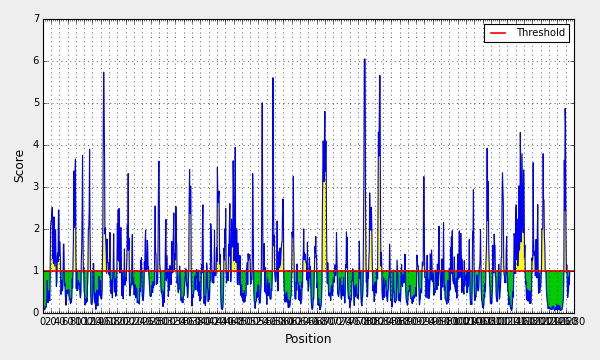


**b**


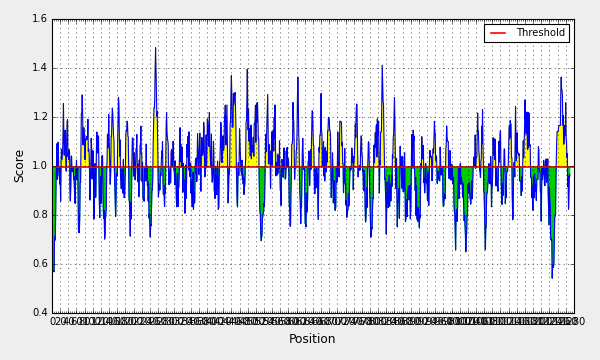


**c**

**Figure S1a-S1c**: Prediction of antigenic determinants. **a**. Kolaskar and Tongaonkar antigenicity scale. **b**. Emini surface accessibility **c**. Chou and Fasman beta turns. Green regions under the threshold color denotes unfavourable related to the properties of interest. Yellow colours are above the threshold sharing higher scores. Horizontal red lines represent the threshold.

**Physiochemical analysis of the SARS-CoV-2 Spike Glycoprotein**

The sequence of the primary structure of SARS-CoV-2 spike glycoprotein was computed, analyzed and tabulated **[Table 1].** The molecular weight was estimated at 141178.47 Da. To calculate the extinction coefficient, wavelengths of varying amount (276, 278, 279, 280 and 282 nm) were computed. But wavelength at 280nm is usually favored because of high protein absorption. So, the extinction coefficient at 280nm is 148960 M^-1^cm^-1^ with respect to the cysteine, trypsin and tyrosine concentrations. The spike glycoprotein is highly stable as the instability index was 33.01, because protein instability index at or above 40 is considered not stable. The isoelectric point or computed theoretical pI of the spike glycoprotein was acidic, lower than 7. The information on the theoretical pI is useful in developing buffer system for the purification of recombinant protein. The total number of negatively charged residues (Asp + Glu) is 110, while that of positively charged residues (Arg + Lys) is 103. The positively charged residues are lesser than the negatively charged counterparts which signifies that the protein is intracellular. The half-life of the protein is 30hours, while the aliphatic index which is the relative volume occupied by aliphatic side chains such as valine, isoleucine, alanine and leucine, is 84.67. At such value, the protein has a high thermostability. The Grand Average hydropathy (GRAVY) of a protein is calculated as the sum of hydropathy values of all amino acids, divided by the number of residues in the sequence. The gravy value of the spike glycoprotein is -0.079, depicting its hydrophilic nature, and better interaction with water. The individual amino composition of the protein is summarized [**Figure 1**]. Every individual amino residue plays a role in the protein function, structure and signaling, depending on their position. The four major amino residues were leucine, serine, threonine and valine. Serine and threonine majorly perform the phosphorylation function which is expedient for the protein’s signaling pathway, as they have hydroxyl functional group with affinity for phosphate group. The least amino residues were Trp (0.90%), Met (1.10%) and His (1.3%).

**S-Table 1**: Physicochemical properties of SARS-CoV-2 Spike glycoprotein

| **Organism** | **m. wt.** | **Seq. length** | **Pi** | **EC (assuming all pairs of Cys residues rom cysteine)** | **EC (assuming all cys residues are reduced)** | **Half-life (hrs)** | **II** | **Gravy** | **-R** | **+R** | **AI** |
| --- | --- | --- | --- | --- | --- | --- | --- | --- | --- | --- | --- |
| SARS-Cov-2 | 141178.47 | 1273 | 6.24 | 148960 | 146460 | 30 | 33.01 | -0.079 | 110 | 103 | 84.67 |

**NB**: (m.wt.: molecular weight, pI: isolectric point, EC: extinction coefficient at 280nm, -R: number of negatively charged residues, +R: number of positively charged residues, Gravy: Grand average hydropathy, AI: Aliphatic index, II: instability index)

**Figure S2**: Percentage of amino acids present in SARS-CoV-2 Spike glycoprotein.


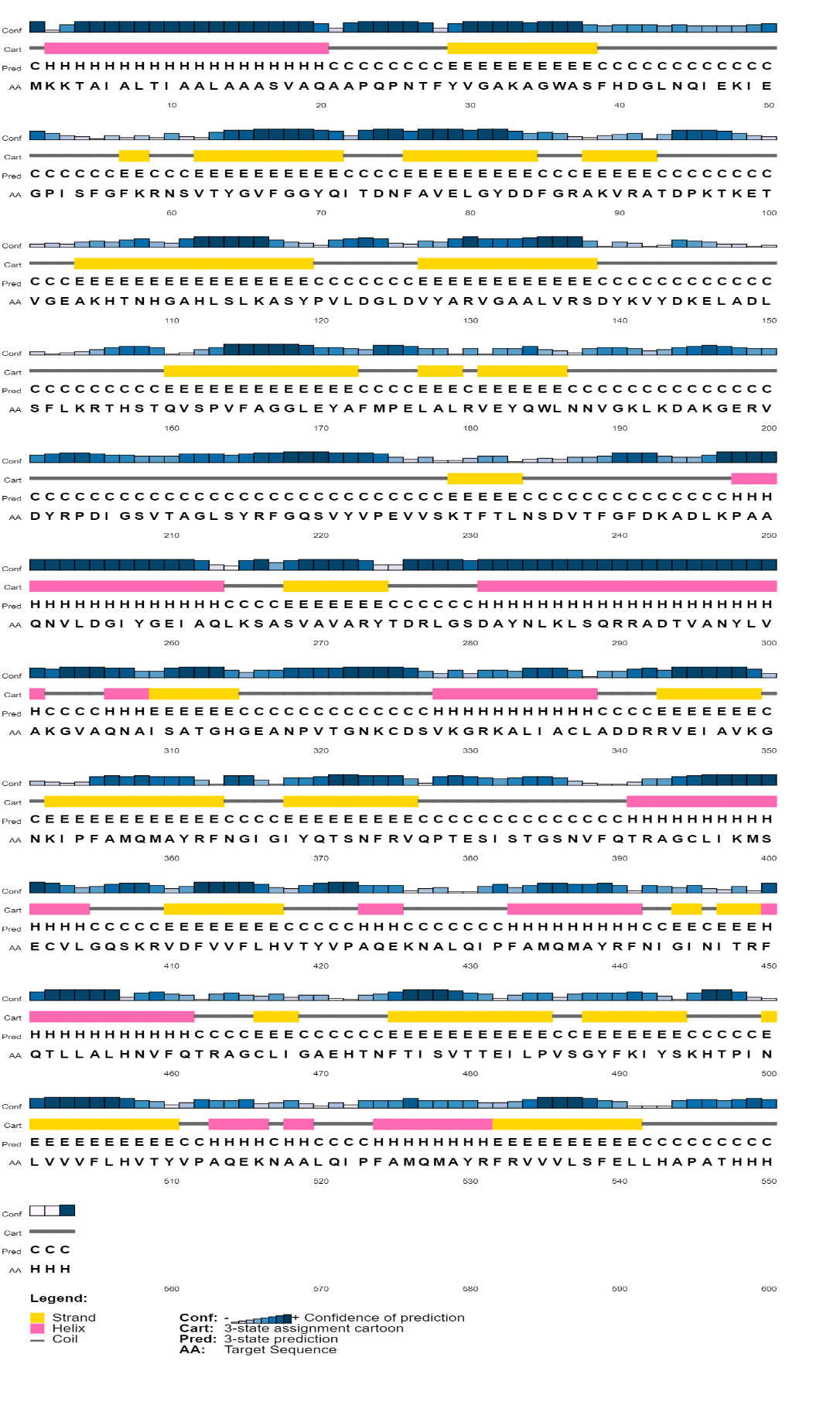


**Figure S3**: Diagrammatic representation of secondary structure prediction of the vaccine construct. Here, the β-strands, α-helix and random coils are indicated by yellow, pink and blue colour, respectively.


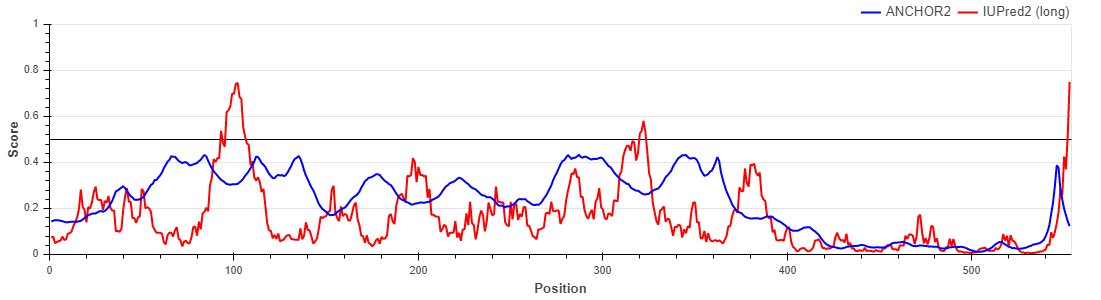


**Figure S4**: Disordered region of the peptide vaccine construct. Regions (lines) exceeding the threshold of 0.5, was considered disordered.
